# Trends in Orthopedic Surgery in Chile: Analysis Between 2004 and 2020

**DOI:** 10.7759/cureus.15080

**Published:** 2021-05-17

**Authors:** Maximiliano Barahona, Diego de los Santos, Nicolas Diaz, Cristian Barrientos, Carlos A Infante

**Affiliations:** 1 Orthopaedic Department/Knee Surgery, Clinica Bupa Santiago, Santiago, CHL; 2 Orthopaedic Department, Hospital Clinico Universidad De Chile, Santiago, CHL; 3 Orthopaedic Department, Hospital Clínico San José, Santiago, CHL; 4 Orthopaedic Department, Clínica Santa María, Santiago, CHL; 5 Orthopaedic Department, Clínica Las Condes, Santiago, CHL

**Keywords:** history, chile, orthopedics, inequalities, public health

## Abstract

Purpose

To describe the trends of orthopedic surgery in Chile since 2004 in terms of the number and gender of surgeons, the incidence of procedures per 100,000 inhabitants (IR), and access by health insurance and type of health center.

Methods

A cross-sectional study was designed. Three databases were analyzed: the free access database of the Chilean Department of Statistics and Health Information (DEIS), which had information on all procedures performed in health institutions in Chile from 2004 to 2020. Then, the orthopedic surgeon registry was requested from the National Superintendence of Health (NSH). Finally, the database of the Chilean Society of Orthopaedic Surgeons (SCHOT) was analyzed. Spearman's correlation was used to determine significant trends during the analyzed period.

Results

The NSH reported 1770 orthopedic surgeons in 2020; 56% were affiliated with SCHOT. An upward trend in the proportion of female orthopedic surgeons was found, from 4.8% in 2004 to 7.6% in 2020. Since 2004, the IR of orthopaedic surgeries has been increasing significantly in both health insurances; the growth in public insurance follows a linear model (R^2^ = 0.970) of parameters β_0_ = - 55982.6 (p <0.000) and β_1_ = 28.02 (p <0.000) while in private insurance, the growth is also linear (R^2^ = 0.890) but with a greater slope: β_0_ = - 104136 (p <0.000) and β_1_ = 52.15 (p <0.000). A significant downward trend was found in the proportion of surgeries performed in the public health network (rho = -0.797, p = 0.0002).

Conclusions

There is a significant increase in the number of orthopedic surgeons and the number of procedures per 100,000 inhabitants. Nevertheless, there is evident inequity in access to orthopedic surgery in Chile and low gender diversity.

## Introduction

Orthopedics is a medical specialty that aims to diagnose and treat injuries and diseases of the musculoskeletal system. In addition, it promotes injury prevention and health status to delay disease progression. There are reports of different ways of immobilization to treat fractures as old as ancient Egypt and old Greece. Hippocrates described a technique to reduce shoulder dislocation. The modern concept of orthopedics was born from the book "Orthopedia," published in 1741 by Nicholas Andry, a professor from the University of Paris. The term is composed of two Greek words: orthos, which means "straight and free of deformity," and paidios, which means "child." The universal symbol of orthopedics came from the same book, the wooden beam to correct a crooked tree's growth [1].

Two great moments have marked modern orthopedics, first at the end of the 19th century with the development of surgery due to advances in asepsis, antisepsis, and the discovery and application of X-rays to the diagnosis of bone pathology. Second, in the middle of the 20th century, the Arbeitsgemeinschaft für Osteosynthesefragen (AO) was founded, which delineated the principles of fracture treatment and osteosynthesis materials development [2-3].

In our country, it seems to be that orthopedic surgeons started working at emergency departments around 1920. In 1937, the Instituto Traumatológico (Trauma Institute) was founded, being the first institution of its kind in Latin America. In 1949, under the administration of Dr. Ernesto Prieto, the first national meeting was arranged to create the Chilean Society of Orthopaedics. A total of 33 doctors attended that meeting [4-5].

In the Chilean health system, population coverage is provided mainly by two types of insurances: the National Health Fund (FONASA), which provides public coverage, and the Health Insurance Institutions (ISAPRE), which provides private coverage. During the period studied, 72.5% of the Chilean population belonged to FONASA and 17.4% belonged to ISAPRE [6]. There are differences in the profile of the affiliates within both health systems. Only 25% of the Chilean population's wealthiest decile is in public health care, compared to the lowest decile, where 92% are in the public health system [7]. Besides, private insurance has a lower proportion of patients over 60 years of age than public insurance (17%) [7].

Two institutions provide inpatient services: the public health network (PHN) and private health institutions (PHI). PHN attends patients that belong to FONASA. PHI corresponds to a broad spectrum that includes university hospitals, mutual insurance company workers, armed forces hospitals, police hospitals, and centers belonging to investment funds. Some patients who belong to FONASA choose to receive their treatment in PHI at their own expense to avoid long waiting times for surgery.

Chile became a member of the Organization for Economic Co-operation and Development (OECD) in 2010, becoming the first South American country to join this organization. This intergovernmental association, among other purposes, provides a platform to compare policy experiences, identify good practices, and seek solutions to common problems. One area of interest in OECD is health.

The purpose of this study is to describe the trends of orthopedic surgery in Chile since 2004, in terms of the number and gender of surgeons, the incidence of procedures per 100,000 inhabitants (IR), and access by health insurance and type of health center.

## Materials and methods

A cross-sectional study was designed. Three databases were analyzed: the free access database of the Chilean Department of Statistics and Health information (DEIS), the orthopedic surgeon registry of the National Superintendence of Health (NSH), and the database of the Chilean Society of Orthopaedic Surgeons (SCHOT).

**Figure 1:**
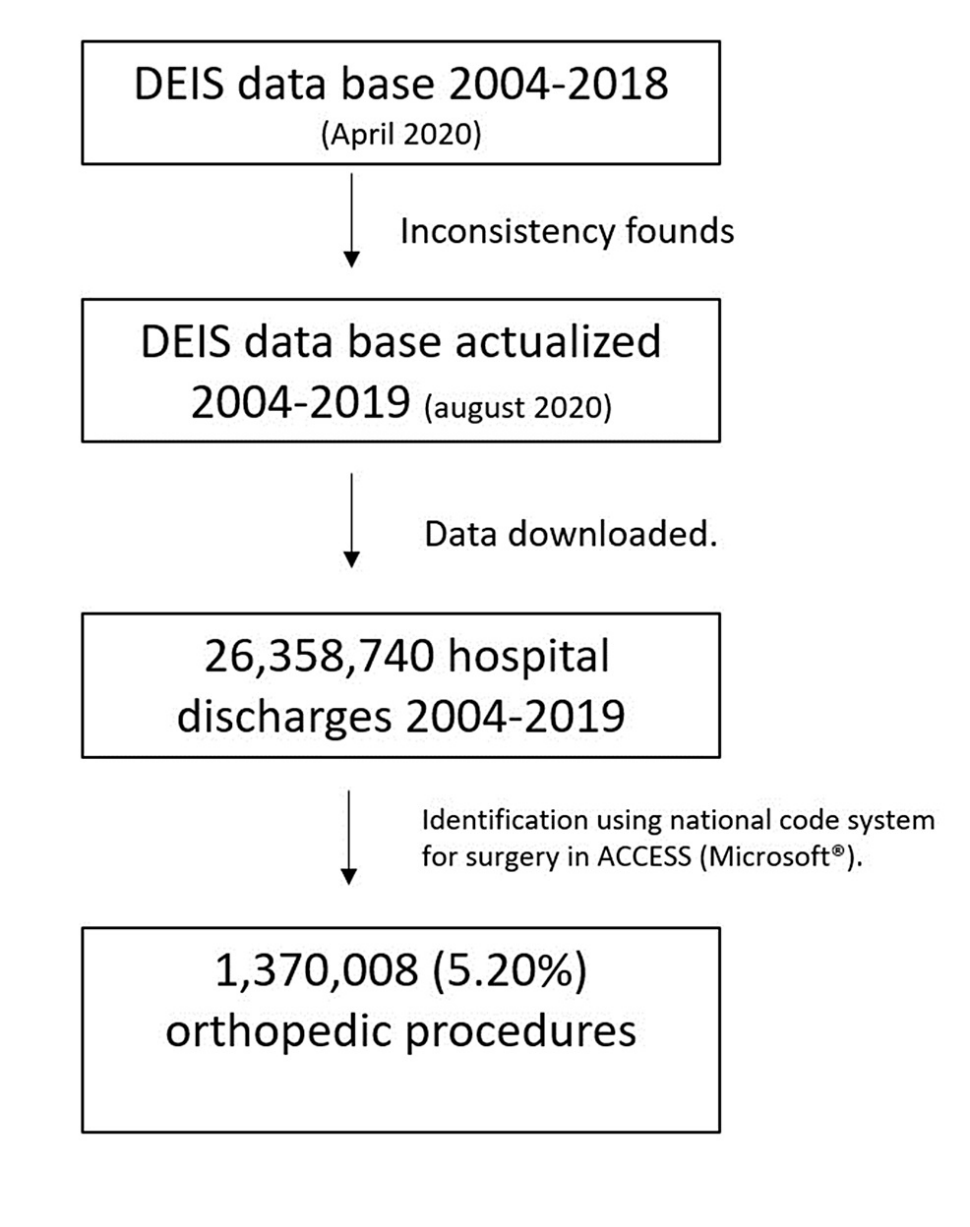
Flow chart of the data extraction from the DEIS database DEIS: Department of Statistics and Health Information (Source: Prepared by the authors from the results of the study)

First, patients who underwent orthopedic surgery between 2004 and 2019 were identified in the free access database of the Chilean DEIS, which depends directly on the Ministry of Health. This register stores all hospital discharges of the country, both from PHN and PHI. During the database analysis, inconsistencies were found, which were reported to DEIS via the National Portal for Transparency in April 2020. The last database update was used - August 11, 2020 (Figure 1). The databases from 2004 to 2019 were downloaded from the DEIS homepage (https://deis.minsal.cl/#datosabiertos). Software Microsoft® Access (Microsoft Corporation, Redmond, WA) was used to manage the data. Patients were identified using the national code for surgeries; all procedures were coded 2104XXX. All orthopedic procedures performed under the "Payment Associated with a Diagnosis" (PAD) program were included. These procedures are coded as follow: 2501030=decompressions of lumbar disk herniations; 2501035=knee meniscectomy; 2501037=median nerve neurolysis; 2501038=r​​​​​​otator cuff repair; 2501039=​​​​​​​ankle fracture fixation; 2501040=​​​​​​​fixations of femoral diaphyseal fractures; 2501041=​​​​​​​fixations of diaphyseal forearm fractures; 2501042=​​​​​​​fixations of diaphyseal humerus fractures; 2501043=​​​​​​​anterior shoulder instability repair; 2501044=​​​​​​​shoulder arthroplasty; 2501045=​​​​​​​treatment of Dupuytren contracture; 2501046=​​​​​​​treatment of hallux valgus; 2501047=​​​​​​​knee anterior cruciate ligament reconstruction; 2501048=​​​​​​​A1 pulley release; 2501049=​​​​​​​treatment of musculoskeletal tumor; 2501050=​​​​​​​wrist/hand synovial cyst resection. PAD corresponds to a program belonging to the public found, known as National Health Found (FONASA). Patients with a selected diagnosis can access a co-pay to undergo surgery in a PHI. Data were exported to STATA v.15 (StataCorp LP, College Station, Texas) for statistical analysis. The incidence rate was calculated per 100,000 inhabitants (IR) using the overall population published in the National Statistics Institute of Chile (INE). Spearman's correlation was used to analyze the trend in the period studied; a significance of 0.05 was used. If the trend was significant, a linear regression was estimated to predict the IR in 2030. The r-squared (R^2^​​​​​^​^)and the parameters β_0_ and β_1_ are reported. After every linear regression, heteroscedasticity and normal distribution of the residuals were evaluated.

Next, the National Health Superintendence database was analyzed and merged with the database of the Chilean SCHOT. The former was requested via the National Portal for Transparency, and the other by a formal letter to the SCHOT board of directors. The number of surgeons, citizens, age, gender, and institution certifying the medical specialty was extracted. All orthopedic surgeons under 65 years were considered "active" since it corresponds to Chile's retirement age. For these two databases, an exploratory analysis was conducted using STATA v.15 (StataCorp LP).

## Results

The National Health Superintendence reported 1770 orthopedic surgeons in 2020; of them, 1669 (94.3%) were Chilean. The proportion of female surgeons was 7.36% (n=134). A total of 1,498 specialists (84.6%) were classified as active on June 30, 2020; the median age was 43 years (range, 28 to 65), and 129 of them were female (8.6%) (Table 1). The metropolitan region gathers 764 specialists (51%), followed by the Valparaíso region with 277 (18.5%) and the Bio-Bio region with 122 (8.2%).

**Table 1:** Shows the number of orthopedic surgeons, the proportions classified as active, the proportion of members of SCHOT, and the percentage of women specialists by year *active surgeon was defined as 65 years of age or less (Source: Prepared by the authors from the results of the study) SCHOT: Chilean Society of Orthopaedic Surgeons

	Orthopaedic surgeons (N)	Actives*	SCHOT members	Women
2004	671	649 (96.7%)	311 (46.3%)	32 (4.8%)
2005	717	695 (96.9%)	337 (47.0%)	34 (4.7%)
2006	762	728 (95.5%)	366 (48.0%)	35 (4.6%)
2007	795	752 (94.6%)	387 (48.7%)	38 (4.8%)
2008	866	808 (93.3%)	402 (46.4%)	39 (4.5%)
2009	945	876 (92.7%)	423 (44.8%)	47 (5.0%)
2010	1003	917 (91.4%)	453 (45.2%)	52 (5.2%)
2011	1061	956 (90.1%)	488 (46.0%)	53 (5.0%)
2012	1119	1000 (89.4%)	519 (46.4%)	61 (5.5%)
2013	1180	1043 (88.4%)	553 (46.9%)	66 (5.6%)
2014	1258	1106 (87.9%)	585 (46.5%)	73 (5.8%)
2015	1331	1163 (87.4%)	629 (47.3%)	87 (6.5%)
2016	1420	1233 (86.8%)	697 (49.1%)	96 (6.8%)
2017	1512	1302 (86.1%)	786 (52.0%)	106 (7.0%)
2018	1613	1380 (85.6%)	877 (54.4%)	115 (7.1%)
2019	1730	1475 (85.3%)	973 (56.2%)	125 (7.43)
2020	1770	1498 (84.6%)	1006 (56.8%)	134 (7.6%)

The certifying entity of the specialty was a university in 986 surgeons (65.8%) while 441 (29%) were via the National Corporation for the Certification of Medical Specialties (CONACEM), 35 (2.3%) were accredited by a health service, 32 (2.14%) accredited by FONASA, and four (0.27%) by the armed forces. The number of members of SCHOT had an upward trend since 2004. Since 2017, SCHOT members represent more than half of the total orthopedic surgeons working in Chile, and in 2020, more than a thousand members (Table 1).

**Figure 2:**
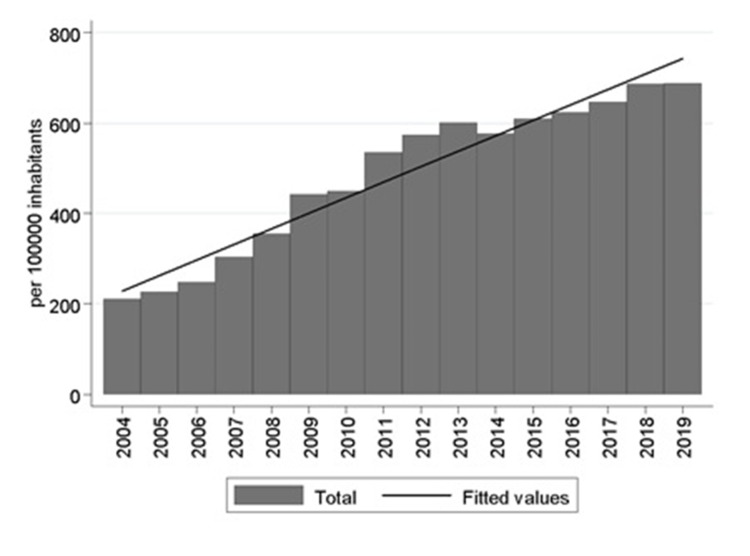
Orthopedic procedure rate per 100,000 inhabitants in Chile between 2004 and 2019 (Source: Prepared by the authors from the results of the study)

The total number of orthopedic procedures in 2019 was 128,735 while in 2004, it was 33,605 (Table 2). The overall proportion of orthopedic procedures during the period studied was 5.2%, and an upward trend was observed, from 2.1% in 2004 to 7.9%, in 2020 (Table 2). The IR has been increasing significantly (rho = 0.99, p <0.000), from 210.0 in 2004 to 687.4 in 2019. This trend followed a linear growth (R2 = 0.942) of parameters, β0 = -68660.49 (p <0.000) and β1 = 34.38 (p <0.000) (Figure 2), which predicts 1,130 procedures per 100,000 inhabitants in 2030.

**Table 2:** Summarized by year the number of total discharges, orthopedic discharges, and incidence rate IR: incidence rate per 100,000 inhabitants; TD: total discharges; OD: orthopedic discharges (Source: Prepared by the authors from the results of the study.)

Year	TD	OD	OD/TD	IR
2004	1,627,748	33,606	2.06%	210.01
2005	1,627,743	36,454	2.24%	225.50
2006	1,637,920	40,442	2.47%	247.62
2007	1,632,888	50,013	3.06%	303.01
2008	1,608,540	59,271	3.68%	355.19
2009	1,682,054	74,655	4.44%	442.35
2010	1,623,875	76,664	4.72%	449.21
2011	1,648,687	92,234	5.59%	534.51
2012	1,670,447	99,879	5.98%	572.54
2013	1,676,936	10,5909	6.32%	600.67
2014	1,660,151	10,2619	6.18%	575.89
2015	1,671,054	10,9778	6.57%	609.66
2016	1,637,265	11,3662	6.94%	624.79
2017	1,637,150	11,8766	7.25%	646.38
2018	1,669,602	12,7321	7.63%	686.28
2019	1,646,680	12,8735	7.82%	687.44
Total	26,358,740	1,370,008	5.20%	

**Figure 3:**
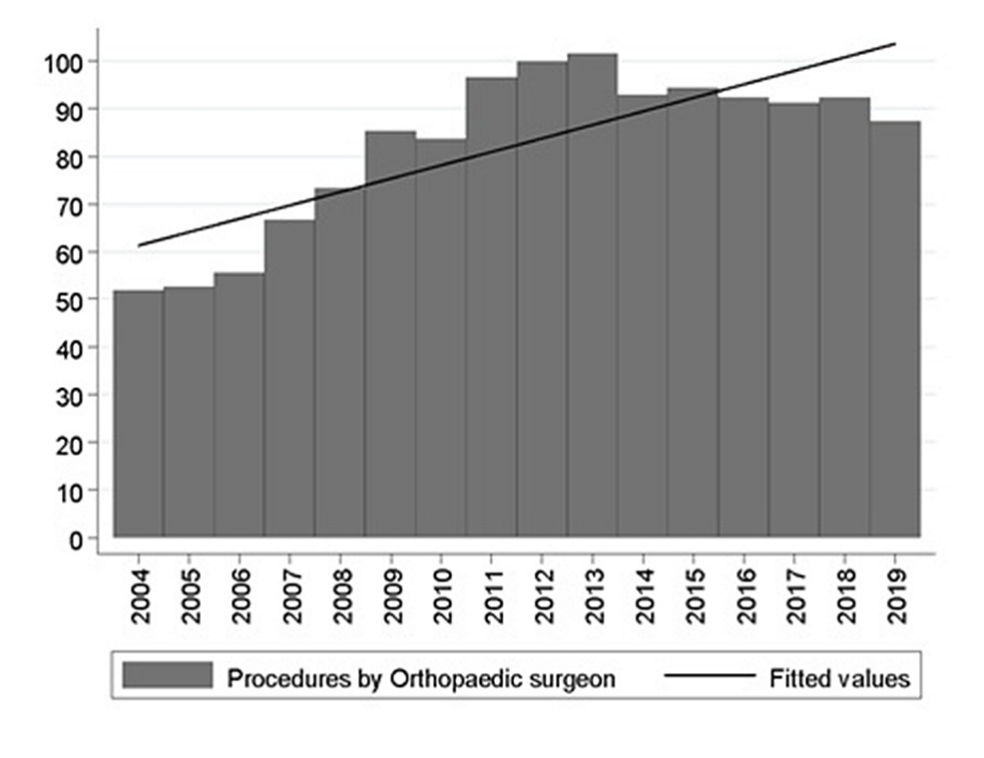
Surgeries rate for each registered and active orthopedic surgeon (Source: Prepared by the authors from the results of the study)

The number of surgeries per active orthopedic surgeon grew between 2004 and 2014, reaching 100 procedures per year. In the following years, a decrease of about 10% was observed, though, in 2019, the rate of surgeries per year was 87 procedures per active orthopedic surgeon (Figure 3).

**Figure 4:**
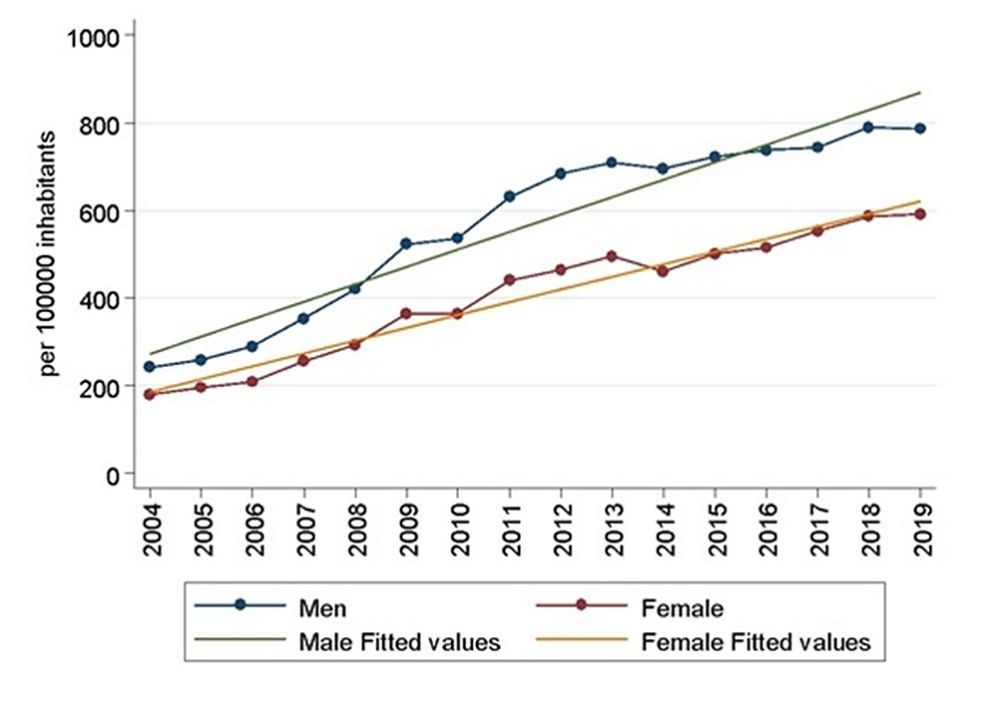
Procedure rate per year per 100,000 inhabitants of each gender (Source: Prepared by the authors from the results of the study)

The average age of patients that underwent orthopedic surgery was 45 years (standard deviation, ±21 years). In 2004, the average age was 43 years (standard deviation, ±23 years) while in 2019, it was 48 years (standard deviation, ±21 years). A sustained growth of the average age is observed (rho = 0.877, p <0.000), which follows a linear behavior (R^2 ^= 0.787) of parameters β_0_ = -370.21 (p <0.000) and β_1_ = 0.207 (p <0.000). A significant upward trend was found in both genders, but male patients were more prone to undergone surgeries among the period studied (Figure 4).

**Figure 5:**
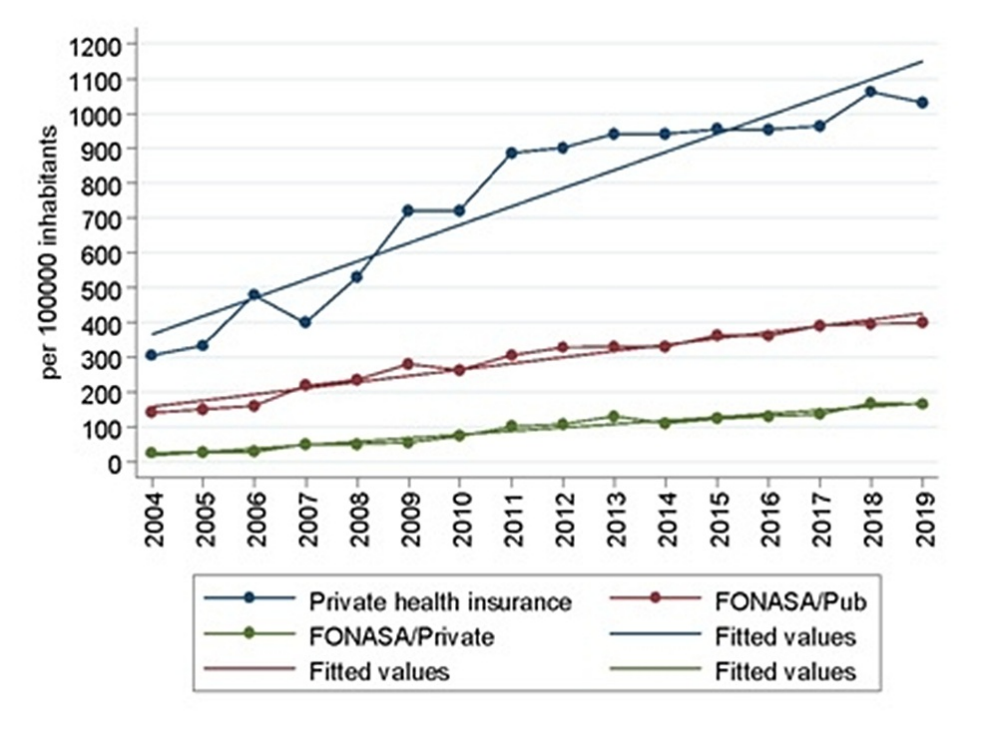
Rate per 100,000 inhabitants belonging to FONASA or ISAPRE who were discharged by an intervention code related to orthopedics. In patients belonging to FONASA, it is segregated by discharge from the center belonging to the public health network (PHN) or private health center FONASA: National Health Found; ISAPRE: Health Insurance Institutions (Source: Prepared by the authors from the results of the study)

The IR of orthopedic procedures has been increasing in both health insurances, FONASA and ISAPRE (Figure 5). In 2004, the IR of patients belonging to ISAPRE was 304.4 while the IR of patients belonging to FONASA was 165.2. Significant growth was observed in both FONASA (rho = 0.997, p> 0.000) and ISAPRE (rho = 0.985; p <0.00). The growth of FONASA patients follows a linear model (R^2^ = 0.970) of parameters β_0_ = - 55982.6 (p <0.000) and β_1_ = 28.02 (p <0.000) and in patients belonging to ISAPRE, growth was also linear (R^2^ = 0.890) but with a greater slope: β_0_ = - 104136 (p <0.000) and β_1_ = 52.15 (p <0.000).

**Figure 6:**
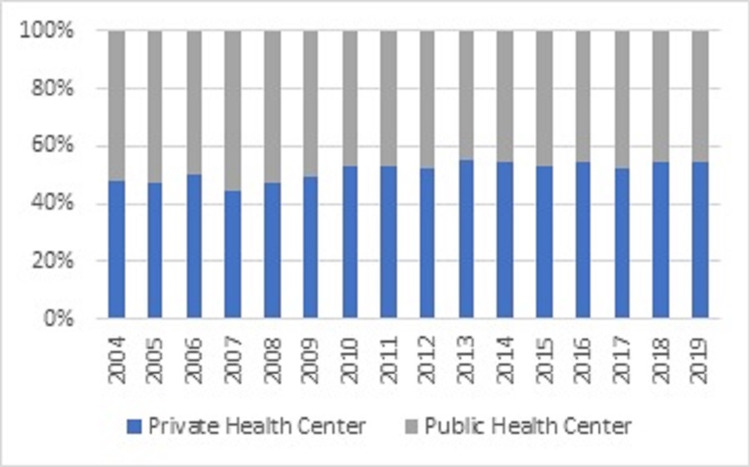
The proportion of hospital discharges between public and private centers (Source: Prepared by the authors from the results of the study)

The number of procedures performed in institutions belonging to the health network in 2004 was 17,370, which represents 51.6% of the procedures. In contrast, 58,730 (45.6%) procedures were performed in 2019 (Figure 6). This downward trend was significant (rho = -0.797, p = 0.0002) and follows a linear model (R^2^ = 0.655) of parameters β_0_ = 11.67 (p <0.000) and β_1_ = -0.01 (p <0.000).

The IR of procedures performed for a fracture in 2019 was 237.8, and 71.39 in 2004. This upward trend was significant (rho = 0.985, p <0.001), following a linear growth (R^2^ 0.906) with parameters β_0_ = - 22616 (p <0.000) and β_1_ = 11.33 (p <0.000). During 2019, the most frequent surgery for fracture per 100,000 inhabitants was the ankle (IR=39.82), followed by the hip (IR=36.77), open fracture (IR=32.14), and distal radius (IR=28.02). The trend of fractures treated in the PHN had a significant downward (rho=-0.8029, p=0.0002); in 2019, only 58% were performed in a public center and was 64% in 2004. The proportion of patients belonging to FONASA that underwent surgery in a private center rose from 8.04% to 18.9% in 2019; this upward trend was significant (rho=0.66, p=0.0002).

**Figure 7:**
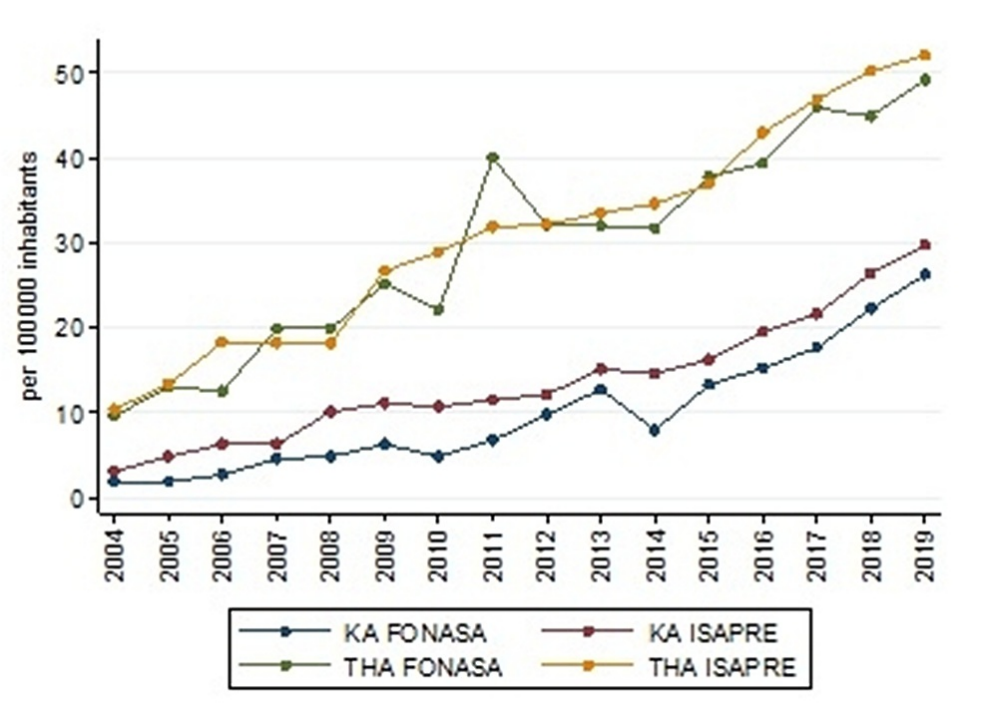
The trend of total hip arthroplasty (THA) and knee arthroplasty (KA) between 2004 and 2019 by type of health insurance (Source: Prepared by the authors from the results of the study)

**Figure 8:**
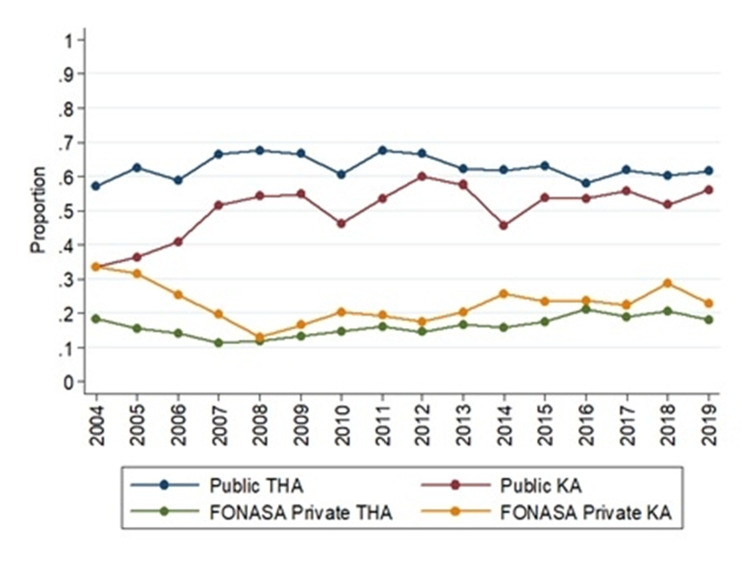
The trend of arthroplasty treated in a public health network and patients belonging to FONASA that underwent surgery in a private health center THA: total hip arthroplasty; KA: knee arthroplasty (Source: Prepared by the authors from the results of the study)

Hip and knee arthroplasty had an upward trend during the period studied. In 2004, knee arthroplasty IR was 2.56, which increased to 28.23 in 2019 (Table 1). This upward trend was significant (rho=0.98, p<0.000). Meanwhile, in total hip arthroplasty (THA), the IR in 2004 was 9.75, and it was 50.20 in 2019. The IR between patients belonging to FONASA and ISAPRE is similar (Figure 7). But adjusted by age, patients over 60 years old belonging to the private insurance underwent 1.8 hips arthroplasties and 2.5 knees arthroplasties more than patients over 60 that belonged to the public insurance. The proportion of hips and knees arthroplasties performed in PHN was around 60%, and 20% of the patients that belongs to FONASA seek medical attention in a private center (Figure 8)

**Figure 9:**
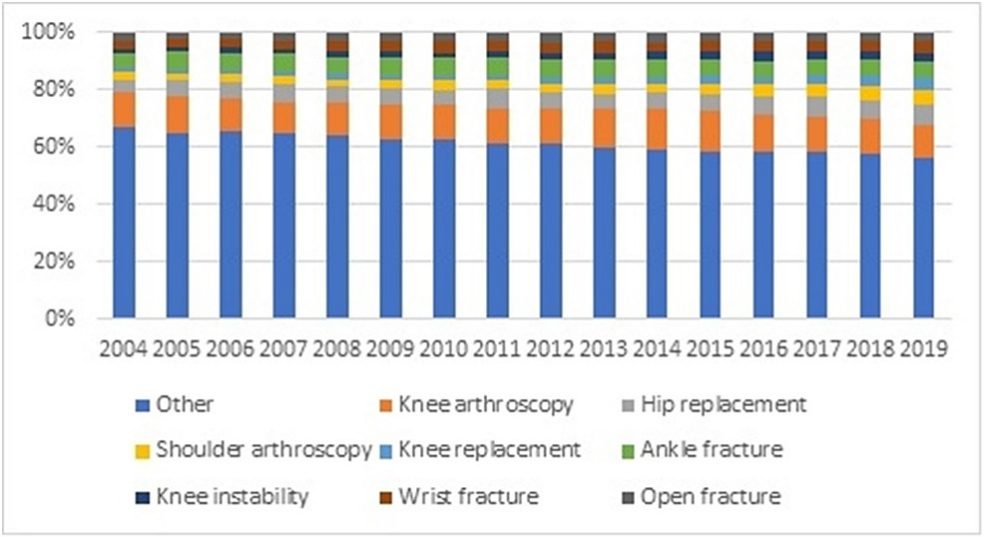
Distribution of the procedures by year (Source: Prepared by the authors from the results of the study)

The most performed procedure was knee arthroscopy (code 2104159/2501035), representing in 2004 and 2019 11.72% and 11.73% of the surgeries, respectively. The second place in 2019 was occupied by hip replacement (codes 2104129/2104229) with 7.30%. Shoulder arthroscopy represents 5.15% of procedures in 2019. In the meantime, total knee arthroplasty, ankle fracture, knee instability, wrist fracture, and complete treatment of open fracture represented 19.92% of the orthopedic surgeries performed in 2019 (Figure 9).

## Discussion

The most significant findings of this study were that in Chile, the rate of orthopedic procedures per 100,000 inhabitants and the number of orthopedic surgeons demonstrated a substantial increase between 2004 and 2019. The number of certified orthopedic surgeons in 2020 was 1.6 times greater than the total number in 2004. This growth is less than reported by the National Health Superintendency for Gynecology and Obstetrics, which increased from 1097 specialists in 2009 to 2969 in 2020. Still, the number of orthopedic surgeons is twice the number of urologists and otolaryngologists.

The density of orthopedic surgeons estimated for 2020 in Chile was 8.6 per 100,000 inhabitants, lower than the 9.3 reported in 2018 in the United States (USA) [8]. The same report shows that in the USA, interstate variability ranges from 7.0 per 100,000 inhabitants in Mississippi to 14.75 in Montana [8]. The information provided by the National Health Superintendency of Chile shows that 51% of orthopedic surgeons reported working in the Metropolitan Region, reaching a density of 10.74 specialists per 100,000 inhabitants. This density falls far below in the most extreme regions of our country, such as the Magallanes Region, in which the density estimate is 2.4. However, the data should be looked at with caution because it is the surgeon's responsibility to keep their current workplace up to date to the Health Superintendency. The biggest problem with this data's bias is that it cannot be used for critical decision-making, especially for the destination of professionals in training programs of medical specialization financed by the state of Chile.

Another highlight found was the low proportion of female orthopedic surgeons, which reached the highest in 2020 (7.6%). Despite the upward trend, the proportion of female orthopedics is far from the 42% of female physicians reported by the Health Superintendency in 2019. Other surgical specialties reported an upward proportion of women. Urology had 0.82% of women in 2009, which rose to 3.57% in 2020. The Health Superintendency reports a more significant increase in Gynecology and Child Surgery, reaching a proportion of 22.7% and 41.1% in 2020, respectively. Diversity is crucial to improve an organization's decision-making and the quality of care provided to patients [9]. One important reason for this gap is the so-called "hidden curriculum," which teaches that orthopedics is a "boys club," that you cannot be a mother and an orthopedic surgeon at the same time, and that the work-life balance is difficult [10]. A survey performed in the USA shows that the most common reason to not consider orthopedic surgery among women was the inability to have a proper balance between life and work (78%) and a lack of solid mentorship in medical school (69%) [11]. The Association of Female Orthopaedic surgeons of Chile was founded in 2019, aiming to prevent discrimination and support female doctors who want to pursue a career as a traumatologist [10]. Adequate gender diversity is considered when the representation is over 30%, not reached in any country. The highest proportions are found in Germany (27%), Estonia (26%), and Spain (25%) while both France (7.1%) and the USA (6.1%) report proportions below Chile [12].

Participation in societies is crucial to share knowledge, keep orthopedic surgeons up to date, and create local guidelines that allow regulation to treat the specialty pathologies. The Chilean Society of Orthopedic Surgeons (SCHOT) is the only related Society of Chile. In 2004, only 28.6% of the orthopedic surgeons belonged to SCHOT; nevertheless, the proportion reached 56.8% in 2020 (Table 1). Among the reasons for this boom is the increasing number of meetings, conferences, training courses, and educational debates that SCHOT had propelled. Also, the Society made an essential change in their selection criteria for new members; before 2012, to be part of the Society, the only way to apply was by presenting and publishing scientific work in the journal of SCHOT, which allows the first author to be a member of the Society. Currently, the requirements for becoming a member of SCHOT is to send the curriculum, two recommendation letters, and an application to the board directory. However, despite efforts, 56.8% represents low community participation compared to other countries; for example, in the USA, 75% of traumatologists belong to one of the four major organizations of the specialty [8].

The number of orthopedic procedures has been increasing at a higher rate than population growth, which is reflected in the linear increase in IR of surgeries. This is a multifactorial phenomenon. The development of new technologies for implants and prostheses, and a lower tolerance to deficits and deformities by the patients, predispose orthopedic surgeons to surgical treatment of the fractures [13]. For example, the development of locking plates and nails with multiple locking options, which allow early motion and immediate weight-bearing, have made into a practically absolute surgery indication distal radius fractures, clavicle fractures, and tibial fractures [14-15]. Moreover, the improvement in prosthesis design and its surgical technique achieving more prolonged survival and better functional outcomes have broadened the indication for joint replacement [16-18]. Life expectancy has increased considerably in the last 20 years, and more patients demand quality years, in which total hip replacement had led the way in the orthopedic solutions [19]. No less significant is the development of related specialties such as anesthesia and intensive care that have made it possible to increase the indications in elderly patients with multiple pathologies that were not previously thought to undergo surgery. As well as the increase of orthopedic surgeons, a crucial reason for the rise of surgical procedures is better hospital infrastructure [20]. Besides, adequately indicated orthopedic surgery has been reported to be cost-effective [21-22]. The growth of orthopedic surgeries in our country becomes evident in its percentage to the total hospital discharge. A sustained increase is shown in this work, going from 2.06% in 2004 to 7.82% in 2019 (Table 2). This fact proves the importance that our specialty has been acquiring in the public health of Chile.

Regarding access, an important bridge was found between the public and private systems. In 2004, patients belonging to the public health insurance - FONASA - had an IR of 165.16; in the meantime, patients that belong to private insurance - ISAPRE - had an IR of 304.38. IR growth up to 1030 and 563.3 for patients belonging to ISAPRE and FONASA, respectively. This gap stayed static across the time studied, representing 1.8 surgeries in ISAPRE patients for every patient who belonged to FONASA.

Despite the gap, IR for arthroplasty has been similar in both insurance organizations. But this has to be looked at with caution. Patients above 60 years, who are at a greater risk of a need for arthroplasty, are concentrated in FONASA. Adjusting by age, the access gap becomes evident, resulting in 1.8 hip and 2.5 knee arthroplasties in the private insurance for every patient over 60 years that belong to public insurance. Moreover, Chile was placed penultimate between OECD countries in the number of hip/knee arthroplasty per 100,000 inhabitants [23]. Although, according to the World Bank, Chile is one of the OECD's younger nations, access to surgical treatment is the main reason to explain the situation. In the last five years, 60% of the arthroplasties were performed in PHN. This proportion is below the United Kingdom and Australia, which reported 80% and 66%, respectively [24-25].

The IR gap between insurance is even more significant in non-fracture or arthroplasty procedures. For example, Chile's most frequent knee arthroscopy procedure represented more than 10% of the orthopedic surgeries. Above 80% of knee arthroscopies were performed in a private institution. No hasty conclusions should be drawn; in France, 70% of the knee arthroscopy was performed in a private institution. The incidence of meniscectomies in the private system was 3.18 times higher than in the public system [26].

The Chilean Ministry of Health has addressed this issue by the PAD program (payment associated with a diagnosis). The beneficiary pays a fixed price that includes all the costs associated with a specific pathology's surgical resolution. PAD was founded in 2014, and a total of 73 pathologies are included, 11 of which are orthopedic procedures [6]. While this is a benefit that can allow access to surgery faster than in the public system and less expensive than in the private sector, it is still a significant expenditure out of the pocket. For example, for knee instability resolution (code 2501047), the co-pay is close to USD 1950 while for meniscectomy (code 2501035), the co-pay is 700 USD [6]. These values are equivalent to 4.68 and 1.68 times the minimum wage of Chile, respectively [7]. Also, PAD has technical barriers; for example, the meniscal repair is not covered, despite the fact that it has been reported to be the standard management for around 20%-25% of meniscal lesions, especially when it is concomitant to an anterior cruciate knee ligament tear [27].

Finally, access to fracture treatment can be analyzed using hip fracture as an example. A recent study shows that patients who belong to FONASA had a greater hip fracture incidence [28]. Moreover, patients treated for hip fracture in the PHN had a higher risk to underwent non-surgical treatment, longer hospital stay, greater in-hospital mortality, and higher one-year mortality as compared to patients treated in a private institution (even if they had public insurance [28-29].

Chile is one of the most unequal countries among the OECD [23,30]. Unfortunately, the health system is not safe for this issue; neither is orthopedics [31]. Inequality for health access cannot be ignored, especially since it has been at the forefront of our country since the social blast that began in October 2019, in which access to health care has been one of the main demands [32].

The Explicit Guarantees of Health (GES) is the most critical public health policy of this century in Chile. GES was implemented in 2006, a set of benefits guaranteed by law allowing access, opportunity, financial protection, and quality of care in a designated list of diseases. Only hip replacement for patients above 65 is the current orthopedic surgery guaranteed by GES for the adult population [33]. Public policies like GES should embrace more orthopedic procedures like hip fracture and knee arthroplasty.

The limitations of this work are those of a country without a national registry of trauma procedures. Other countries have implemented national registries for certain sentinel surgeries such as hip and knee prostheses, cruciate ligament reconstruction, and hip fracture [34-36]. This information has been extremely useful in improving the quality of care and patient satisfaction [37]. Another limitation is that some procedures performed by orthopedic surgeons could have been registered under a neurosurgery-related code as lumbar spine surgery or median nerve neurolysis. The former is a significant reason why Chile urgently requires modernization of the surgical coding system and national registries. Also, nowadays, is not possible to differentiate a total knee replacement from unicompartmental knee arthroplasty, as both procedures use the same code (2104153), which could also be solved by a modernization of the surgical code system.

## Conclusions

The last 16 years of orthopedic surgery in Chile can be described in two different ways. On one side, the glass could be viewed as half full: the number of procedures per 100,000 inhabitants and the number of surgeons had grown fast, which has allowed treating a significant number of pathologies. But on the other side, the glass is half empty: the access to orthopedic procedures had been as unequal as other areas of Chile. Also, gender diversity had undergone low growth.

The Chilean Society of Orthopaedic Surgeons has to face critical challenges: to increase the number of members, promote gender diversity, and encourage the development of national registries and guidelines for good clinical practice that force the improvement of public health policies.

## Appendices

Availability of data

The datasets used and/or analyzed during the current study is available on the DEIS homepage https://deis.minsal.cl/#datosabiertos. The database of the Health Superintendency is available at the request of any citizen via the National Portal of Transparency.
